# Mobile App-Guided Home Exercise Versus Conventional Pamphlet-Based Instruction After Corticosteroid Injection in Patients with Frozen Shoulder: A Pilot Randomized Trial

**DOI:** 10.3390/healthcare14162647

**Published:** 2026-08-20

**Authors:** Yu-Jia Sheu, Chien-Hung Liao, Yu-Wei Hsieh, Chia-Ying Chung, Chih-Chi Chen

**Affiliations:** 1 Department of Physical Medicine and Rehabilitation, Chang Gung Memorial Hospital, Chang Gung University, Taoyuan 333423, Taiwan; 2 Department of Trauma and Emergency Surgery, Chang Gung Memorial Hospital, Chang Gung University, Taoyuan 333423, Taiwan; 3 Department of Occupational Therapy and Graduate Institute of Behavioral Sciences, College of Medicine, Chang Gung University, Taoyuan 33302, Taiwan

**Keywords:** frozen shoulder, adhesive capsulitis, triamcinolone acetonide, exercise therapy, telerehabilitation, mobile applications, pilot projects

## Abstract

**Highlights:**

**What are the main findings?**

- App-assisted telerehabilitation facilitates significantly greater improvements in shoulder abduction and internal rotation compared to pamphlet-assisted home exercise.
- Home exercise programs improved pain, function, and shoulder range of motion over the 4-week rehabilitation period.

**What are the implications of the main findings?**

- Mobile app-guided home exercise is an effective adjunct to corticosteroid injection during early frozen shoulder rehabilitation.
- Digital telerehabilitation enables objective monitoring of exercise adherence without relying solely on patient self-report.

**Abstract:**

**Background**: Frozen shoulder (FS) is a debilitating condition characterized by pain and restricted range of motion (ROM), often leading to prolonged treatment and economic burden. Home exercise programs are effective for FS, so we developed the Defrozen app to support home exercise. This randomized controlled trial (RCT) compared app- versus pamphlet-assisted home exercise programs in patients with FS. **Methods**: In this RCT, twenty-one patients with FS who received corticosteroid injections were randomized (2:1) to app or pamphlet-assisted home exercise. Baseline assessments were performed, and all participants initiated their assigned home exercise program, which lasted 4 weeks. Outcomes included shoulder ROM, pain intensity (numeric rating scale, NRS), and functional outcomes (Oxford Shoulder Score [OSS] and Quick Disabilities of the Arm, Shoulder, and Hand [QuickDASH] score). **Results**: Both groups demonstrated significant within-group improvements in ROM measures, NRS, OSS, and QuickDASH scores (*p* < 0.05). Between-group comparisons using analysis of covariance (ANCOVA) showed significantly greater gains in shoulder abduction (*p* = 0.023) and internal rotation (*p* = 0.020) in the app-assisted group compared with the pamphlet group. **Conclusions**: A four-week app-based home exercise program provides additional benefits in improving shoulder ROM compared with conventional pamphlet-based instruction in FS after corticosteroid injection.

## 1. Introduction

Frozen shoulder (FS), also known as adhesive capsulitis, is characterized by a thickened and fibrosed joint capsule, joint contraction, and reduced intra-articular volume [1]. Recent population-based studies in adults aged ≥65 years reported a one-year prevalence of approximately 0.35–0.4% and a lifetime prevalence of 2.4% [2]. Although FS is generally considered a self-limiting condition with a natural course of one to three years, nearly half of patients experience residual symptoms beyond this period [3]. Pain and limited range of motion (ROM) can severely impair activities of daily living (ADL), reduce productivity, and impose a substantial economic burden on patients’ families and society [4,5]. Given the prolonged disease course and associated economic burden, timely and cost-effective treatment strategies are essential.

Current management of FS focuses on alleviating pain and restoring shoulder ROM and function [6]. Shoulder exercises are widely regarded as effective, particularly for improving mobility, reducing stiffness and pain, and enhancing function [7]. A recent meta-analysis showed that incorporating exercise into treatment programs yields better ROM outcomes than non-exercise approaches. In contrast, the specific type of exercise, as ROM exercises, wall exercises, scapular exercises, or pendulum exercises, appears to make limited difference [7]. The systematic review of 65 studies involving 4097 patients presented that those with symptoms lasting less than one year achieved superior outcomes when intra-articular corticosteroid injections combined with home exercise, comparing with injections alone [8]. These interventions are most indicated during the painful–stiff phases (the freezing or early frozen stages), when pain and limitations in ROM are most pronounced.

Mobile health (mHealth) technologies have recently gained attention as effective tools to support patient self-management in medical care [9,10]. These platforms, recognized by the World Health Organization as components of digital health [11], include smartphone applications that can deliver exercise demonstrations and monitor disease progression [9,10]. A growing body of literature supports the role of mobile apps in enhancing outcomes for musculoskeletal conditions, including chronic back pain and arthritis [12]. The COVID-19 pandemic has further accelerated the adoption of telerehabilitation and mobile solutions in musculoskeletal care. In response to this need, we developed the Defrozen app, a user-friendly mobile application that delivers seven shoulder exercises covering all major directions of shoulder movement, including flexion, extension, abduction, adduction, internal rotation, external rotation, and pectoral muscle stretching. The app provides exercise demonstrations to ensure correct performance, automatically records whether each session is completed, and offers a calendar view so users can visualize their daily and cumulative exercise frequency. The design emphasizes simplicity and usability to facilitate patients’ adherence to home exercise programs. A prior feasibility study confirmed the high usability and user acceptance of the Defrozen app [13].

Furthermore, evidence of rehabilitation in frozen shoulder also goes beyond digitally delivered intervention. Deodato et al. used inertial measurement and pressure pain threshold assessment to objectively evaluate improvements in shoulder flexion and abduction and pressure pain thresholds after physiotherapy programs [14]. Lin et al. found that proprioceptive neuromuscular facilitation (PNF) as an adjunct to conventional rehabilitation improved pain and range of motion, and was associated with reductions in MRI-measured coracohumeral ligament and axillary recess capsule thickness [15]. Both studies showed that the effects of rehabilitation delivered in frozen shoulder can be evaluated by complementary clinical, biomechanical, and imaging outcomes.

This randomized controlled trial was designed to compare the clinical outcomes of a Defrozen app-guided home exercise program with those of a conventional pamphlet-based program in patients with FS. Moreover, the trial is expected to provide evidence to inform future confirmatory studies and clinical practice. We specifically aimed to determine whether app-based intervention provides greater improvements in shoulder ROM, pain, and functional outcomes.

## 2. Materials and Methods

### 2.1. Study Design

This assessor-blinded, randomized controlled trial aimed to evaluate the effects of a mobile app-assisted home exercise program compared with a traditional pamphlet-based program for four weeks on improving shoulder ROM in patients with adhesive capsulitis (frozen shoulder). A total of 21 participants were enrolled in this pilot trial. The study was conducted across three hospitals in northern Taiwan between 11 September 2022 and 31 December 2023. Ethical approval was obtained from the Chang Gung Memorial Hospital Institutional Review Board. This study was registered at ClinicalTrials.gov (NCT05980572); the present report represents the preliminary pilot phase of the registered trial. The randomization protocol used a 2:1 allocation ratio to prioritize intervention group recruitment for app usability analysis, which was pre-specified in the internal feasibility planning [13]. Given the pilot nature of this study, no formal a priori sample size calculation was performed, and the findings should be interpreted as exploratory.

### 2.2. Participants

Adults aged 40 years and older with a diagnosis of unilateral adhesive capsulitis who were scheduled to receive corticosteroid injection in the outpatient departments were considered eligible. The clinical diagnostic criteria for adhesive capsulitis included a history of persistent pain and stiffness for at least one month and at least a 30% restriction in two or more directions of shoulder movement (abduction, flexion, internal rotation, or external rotation) [16]. Patients with bilateral frozen shoulder, a history of significant shoulder trauma, prior upper limb surgery, or corticosteroid injection within the preceding three months were excluded, as were those with cognitive impairments or inability to use a smartphone.

### 2.3. Interventions

Following enrollment and baseline assessment, all participants received standardized corticosteroid injections prior to starting the home exercise program. Injections were administered to both the glenohumeral joint and the subacromial space, based on prior evidence suggesting synergistic benefits [17]. Each site received 1 mL of triamcinolone acetonide (20 mg/mL) mixed with 1 mL of 1% lidocaine [18]. Injections were performed under ultrasound guidance by an experienced physiatrist to ensure accurate placement. Immediately afterward, all participants received standardized home exercise instruction from the same physical therapist.

**Figure 1:**
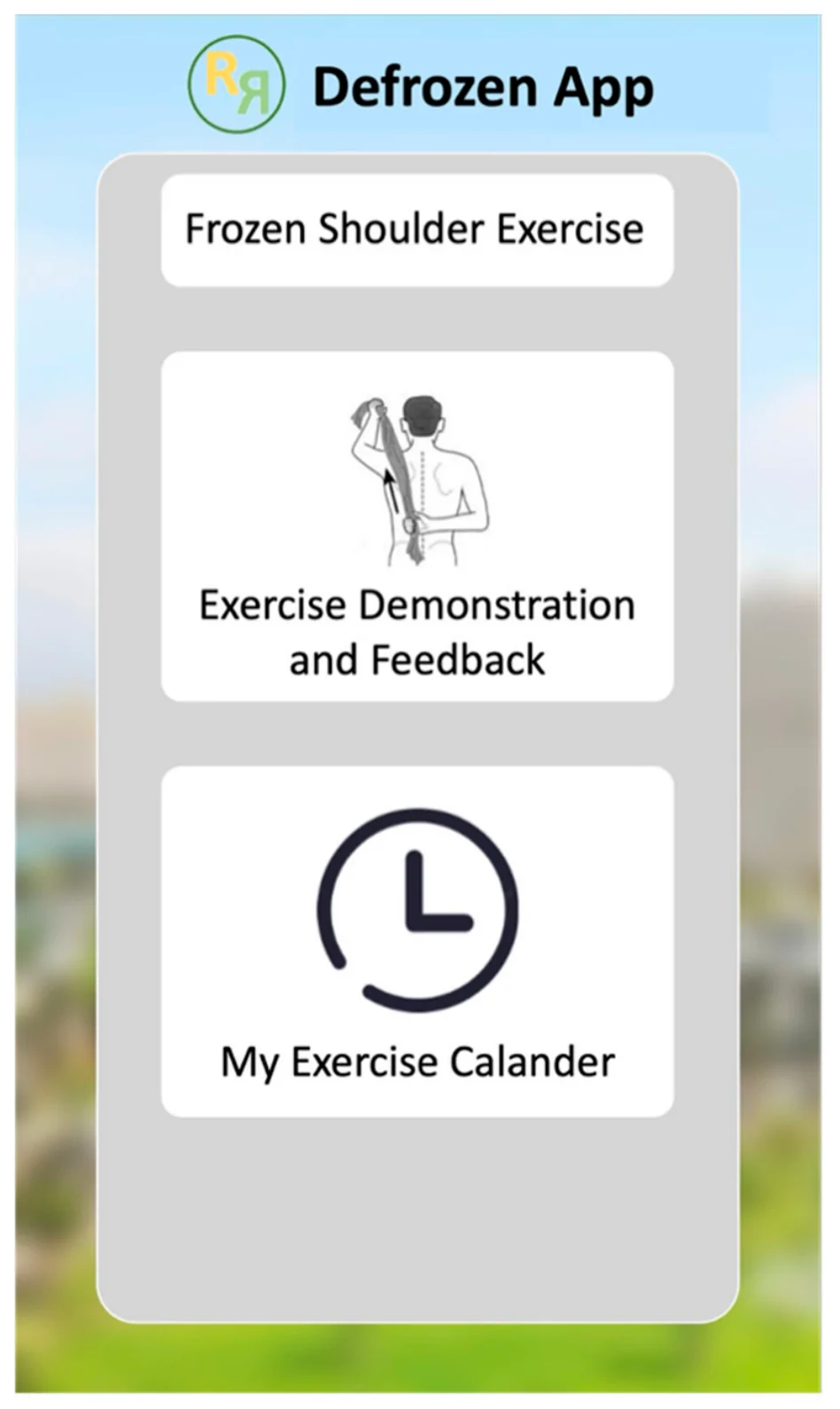
User interface of the Defrozen app, showing the modules for exercise demonstration and progress calendar.

The home exercise program began on the second day after injection, and participants were randomly assigned to either the experimental group (Defrozen app-assisted exercise) or the control group (pamphlet-based exercise). Participants in the experimental group were instructed to follow a structured daily home exercise program using the Defrozen mobile app [13]. The app provided exercise demonstrations to ensure correct performance, automatically recorded whether each session was completed, and offered a calendar view so users could visualize their daily and cumulative exercise frequency (Figure 1).

**Figure 2:**
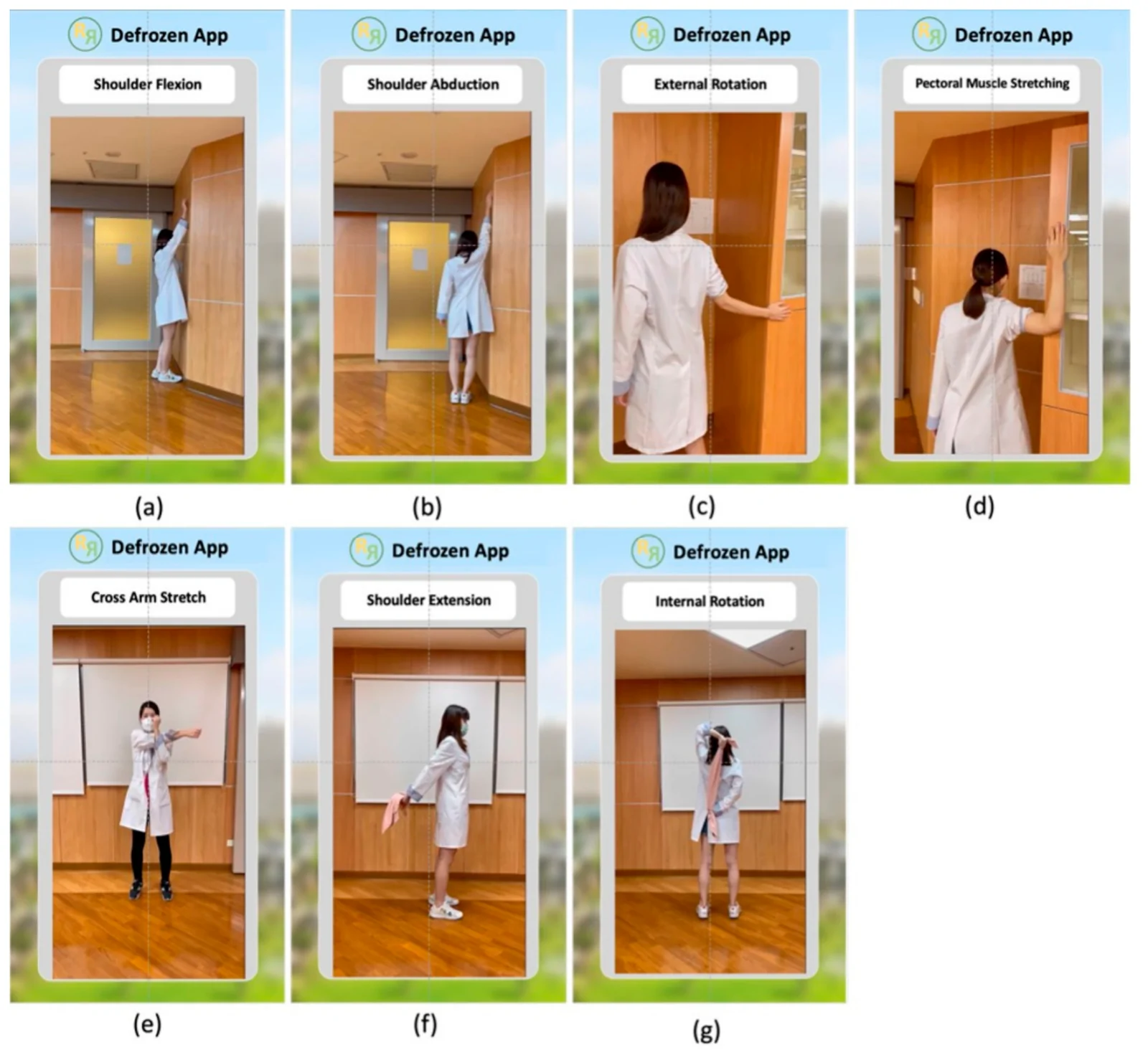
Illustrations of the seven shoulder exercises included in the Defrozen app: (**a**) finger-walk for shoulder flexion; (**b**) finger-walk for shoulder abduction; (**c**) self-stretch for external rotation; (**d**) self-stretch for pectoralis muscle; (**e**) cross-arm stretch for adduction; (**f**) towel-assisted stretch for extension; (**g**) towel-assisted stretch for internal rotation.

The design emphasized simplicity and usability to support consistent exercise engagement. The control group performed the same set of shoulder exercises using printed pamphlets with illustrated instructions. Both groups received standardized guidance at baseline. The exercise protocol consisted of seven movements: shoulder flexion, shoulder abduction, shoulder extension, shoulder adduction, shoulder internal rotation, shoulder external rotation, and pectoral muscle stretching (Figure 2a–g). Our app videos showed the complete movement sequence from the starting to the ending position. These exercises were selected to comprehensively target all major directions of shoulder movement, as commonly recommended in frozen shoulder rehabilitation. Each exercise was performed for ten repetitions per set, with all seven exercises prescribed to be completed three times daily. No progression in exercise difficulty or dosage was applied during the four-week intervention, and participants followed the same exercise program throughout the study. Participants were reminded to follow their assigned exercise protocol for four consecutive weeks.

### 2.4. Outcomes

Outcome data were assessed at baseline and after four weeks of intervention. Outcome measures included shoulder ROM, shoulder pain intensity, and self-reported functional scores.

#### 2.4.1. Range of Motion (ROM)

Shoulder ROM was measured using a universal goniometer with the participant in an erect, neutral position. Passive ROM for flexion, extension, abduction, internal rotation and external rotation was recorded following established protocols [16,19]. Each angle was measured three times, and the mean was recorded [20].

#### 2.4.2. Shoulder Pain Intensity

Pain intensity was assessed using the 11-point Numerical Rating Scale (NRS), where 0 indicates no pain and 10 indicates the worst imaginable pain [21].

#### 2.4.3. Oxford Shoulder Score (OSS)

The OSS is a 12-item patient-reported questionnaire assessing shoulder pain and function in individuals with shoulder pain, with scores ranging from 0 (worst) to 48 (best) [22]. It has been employed to evaluate treatment effectiveness in cases of frozen shoulder, and the validated Taiwanese version was used in this study [22,23,24,25].

#### 2.4.4. Quick Disabilities of the Arm, Shoulder, and Hand (QuickDASH) Score

The QuickDASH is an 11-item patient-reported measure of upper limb disability, producing scores from 0 (no disability) to 100 (most severe disability) [26,27]. The validated Taiwanese version was used for assessing the health status of our patients [28].

### 2.5. Adherence

All participants were instructed to complete the full sequence of seven exercises (shoulder flexion, shoulder abduction, shoulder extension, shoulder adduction, shoulder internal rotation, shoulder external rotation, and pectoral muscle stretching) three times daily for four weeks. Completion of all seven exercises was counted as one completed exercise session. In the experimental group, each completed session was automatically recorded by the app. In the pamphlet group, participants manually recorded the number of completed sessions in a daily exercise log. Exercise adherence was quantified as the mean number of completed sessions per day.

### 2.6. Randomization and Blinding

Participants were randomly assigned to either the app-assisted exercise group or the control group using a computer-generated randomization sequence with a 2:1 allocation ratio. The sequence was prepared by a study team member who was not involved in recruitment, clinical care, or outcome assessment. Group assignments were concealed in consecutively numbered, opaque, sealed envelopes. After confirming eligibility and obtaining informed consent, the envelope corresponding to the participant’s sequence number was opened to reveal the assignment. Due to the nature of the intervention, participants were not blinded to group allocation. All outcome assessments, however, were conducted by a physician blinded to group assignment and independent of treatment and data analysis.

### 2.7. Statistical Analysis

All analyses were performed using Stata version 14.0 (StataCorp LLC, College Station, TX, USA). Baseline characteristics were compared using independent-sample *t*-tests (continuous) and Fisher’s exact tests (categorical) for all randomized participants. Efficacy analyses were conducted as a complete-case analysis. Within-group changes from baseline to four weeks were analyzed using paired *t*-tests.

Between-group post-intervention outcomes were compared using ANCOVA with baseline values as covariates. For each outcome, change scores (post–pre) were also calculated, and associations with recorded exercise time were explored using linear regression adjusted for baseline and group. For ANCOVA models, standardized effect sizes were reported as partial eta squared (ηp^2^) with 95% confidence intervals to aid interpretation of clinical relevance in this pilot sample.

Given the small sample size and the 2:1 allocation ratio, this study was exploratory, and no formal sample size calculation was performed. All tests were two-tailed with α = 0.05.

## 3. Results

### 3.1. Participants

**Figure 3:**
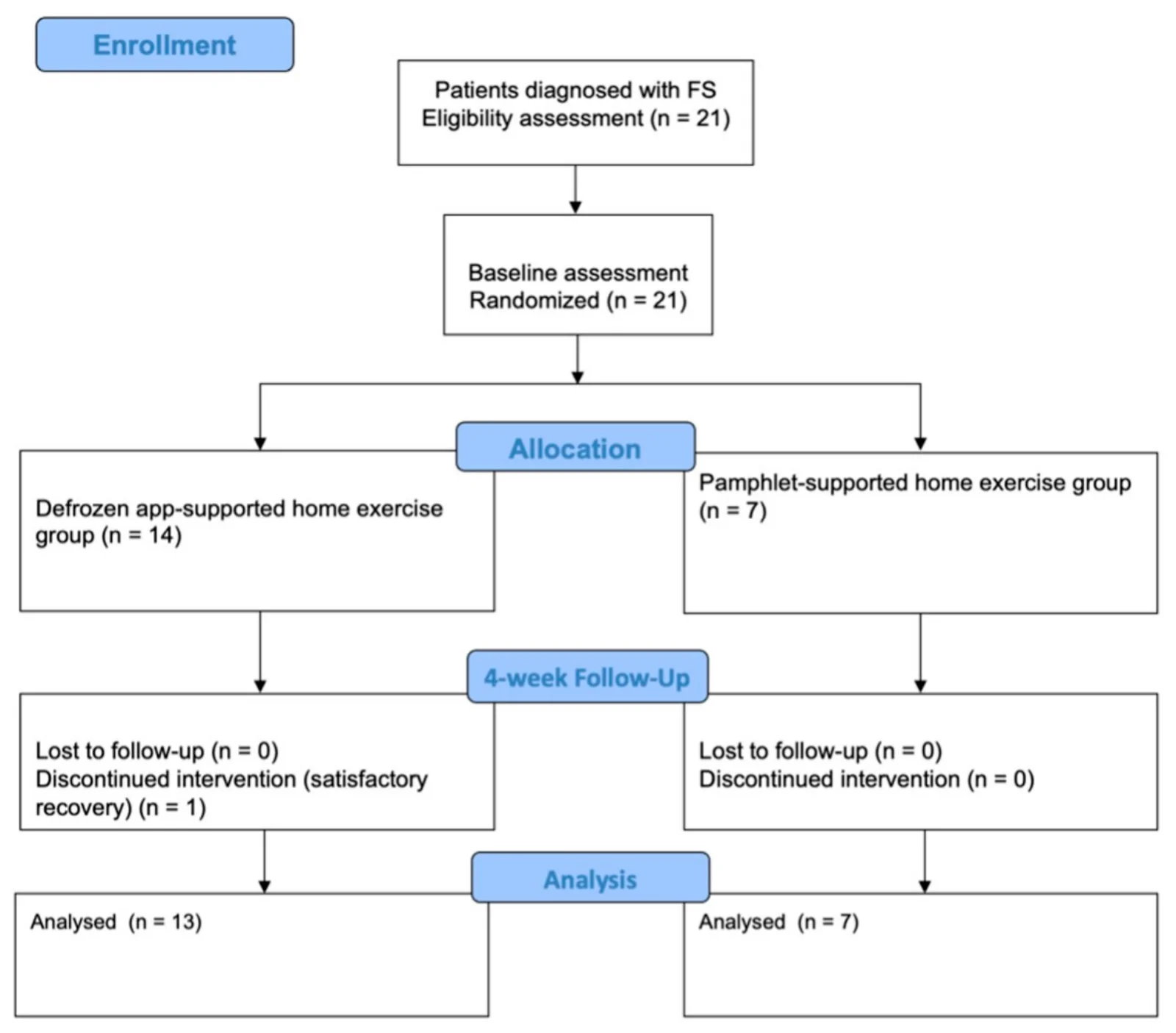
CONSORT flow diagram of participant enrollment, allocation, follow-up, and analysis.

A total of 21 participants were randomized (app group, *n* = 14; pamphlet group, *n* = 7). One participant in the app group discontinued before the four-week visit after reporting satisfactory recovery via telephone follow-up. Twenty participants completed follow-up (app group, *n* = 13; pamphlet group, *n* = 7) and were included in the complete-case analysis. No adverse events were reported (Figure 3).

### 3.2. Baseline Characteristics

There were no significant differences at baseline in terms of age, gender, hand dominance, ROM, NRS, and QuickDASH scores between the two groups. The app group had a higher baseline OSS (*p* = 0.009), indicating better baseline shoulder function (Table 1). These differences were accounted for in the adjusted analyses by including baseline values as covariates.

### 3.3. Within-Group Changes

**Figure 4:**
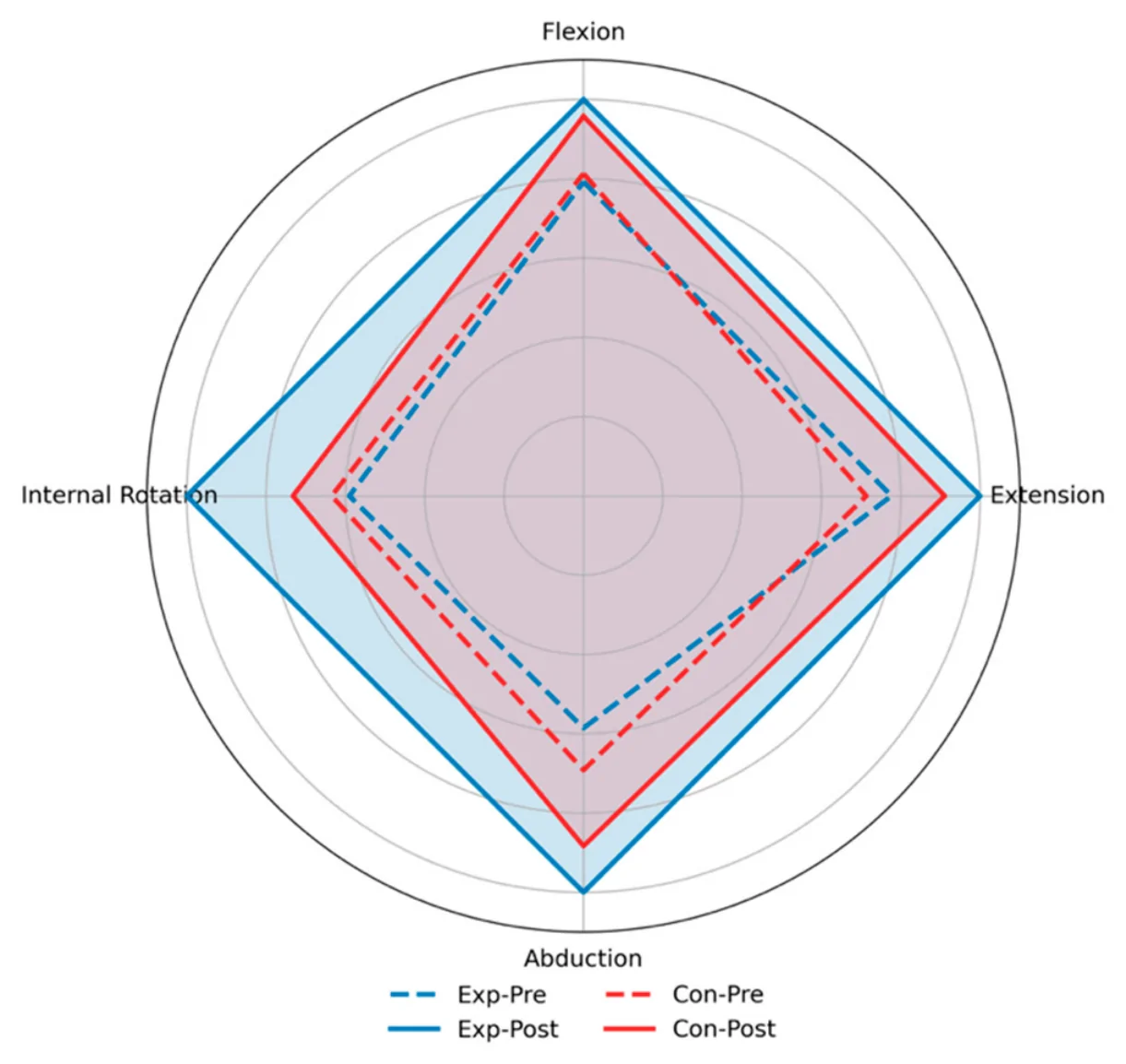
Radar chart comparing shoulder range of motion (ROM) between experimental and control groups. Blue lines represent the experimental group, and red lines represent the control group. Dashed lines denote pre-treatment baselines, while solid lines with shaded areas indicate post-treatment outcomes. Data are normalized across four movement directions: Flexion, Extension, Abduction, and Internal Rotation.

Over four weeks, improvements were observed in both groups across most ROM planes and in pain and function (Table 2). In the app group, significant improvements were observed in flexion, extension, abduction, external rotation, and internal rotation (*p* ≤ 0.014). On the other side, in the pamphlet group, the improvements were observed in flexion, extension, abduction, and external rotation (*p* ≤ 0.037), as Figure 4 presented. In app group, we also found decreased NRS (*p* < 0.001), increased OSS (*p* = 0.006), and decreased QuickDASH score (*p* = 0.003). In the pamphlet group, we found decreased NRS (*p* < 0.001), increased OSS (*p* = 0.002), and decreased QuickDASH score (*p* < 0.001).

### 3.4. Between-Group Comparisons (ANCOVA)

Table 3 presents the results of the analysis of covariance (ANCOVA) assessing between-group differences in post-intervention outcomes, adjusted for baseline values. After adjustment, the experimental group showed significantly greater improvements in shoulder abduction (*p* = 0.023) and internal rotation (*p* = 0.020) compared with the control group. No significant between-group differences were found for flexion, extension, or external rotation, NRS, OSS and QuickDASH score.

### 3.5. Adherence and Exercise Time

Linear models of change scores (post–pre), adjusted for exercise time, baseline, and group, indicated that the baseline severity was the strongest predictor of improvement, with patients with more restricted ROM or higher pain achieving greater gains (Table 4). Exercise time was not a significant predictor in any model. The group effect remained significant for internal rotation (*p* = 0.035) and borderline for abduction (*p* = 0.050), corroborating the ANCOVA findings. Three control participants misplaced paper logs, whereas the app automatically captured session completion.

## 4. Discussion

The current study demonstrated that patients with frozen shoulder who received corticosteroid injections, followed by either Defrozen app-based or pamphlet-based home exercise, experienced meaningful improvements in ROM, pain, and function after four weeks. The app-based intervention was associated with significantly greater gains in shoulder abduction and internal rotation compared with the pamphlet-based program. These findings highlight the potential benefits of app-based telerehabilitation are particularly relevant in the context of increasing reliance on digital health solutions.

Mobile health (mHealth) applications have gained wide acceptance, especially among younger populations, and are increasingly recognized by global health authorities as valuable tools for healthcare delivery [11]. A recent review on telemedicine in musculoskeletal disorders concluded that digital interventions provide non-inferior outcomes, comparable patient experiences, and greater cost-efficiency while improving accessibility [29]. Our findings add to this evidence by suggesting that an app-based approach may specifically enhance selected dimensions of shoulder ROM in frozen shoulder rehabilitation.

Several mobile applications have been developed for the telerehabilitation of musculoskeletal shoulder problems [30,31]. While many studies in frozen shoulder have primarily focused on feasibility, usability, or user acceptance [32,33,34,35], relatively few have investigated clinical effectiveness, and most were not randomized controlled trials. To date, only one randomized controlled trial has evaluated an app specifically designed for frozen shoulder exercises [36]. In that study, Choi et al. developed a program incorporating four movements (shoulder flexion, cross-body adduction, and sleep stretches for internal and external rotation), requiring participants to hold a smartphone during most exercises to receive real-time visual and auditory feedback. The app did not significantly improve pain or ROM compared with conventional home exercise, which the authors attributed to its limited exercise repertoire and incomplete feedback integration.

Shoulder exercises are widely recognized as an effective intervention for improving mobility and reducing stiffness in frozen shoulder, with meta-analyses showing greater ROM gains when exercise is integrated into the treatment program compared to non-exercise approaches [8]. In this study, both groups performed daily home exercises following intra-articular corticosteroid injections and demonstrated meaningful within-group improvements in ROM, pain, and function. As the only difference between groups was the mode of exercise delivery—via the Defrozen mobile application or a paper pamphlet—the observed between-group differences can reasonably be attributed to the delivery method. The app-assisted group achieved significantly greater improvements in abduction and internal rotation, and these gains exceeded the minimal clinically important difference (MCID) of 15° reported by Sharma et al. [16]. The structured video demonstrations and automated recording provided by the app may have supported exercise engagement and execution. However, exercise accuracy and movement quality were not directly assessed; therefore, this explanation remains hypothetical.

Both groups demonstrated significant improvements in functional outcomes, with no significant between-group differences in OSS or QuickDASH scores. It is plausible that the ROM differences, while statistically significant, were not of sufficient magnitude to translate into measurable functional gains within a four-week follow-up period. This observation is consistent with previous large-scale randomized trials, such as the UK FROST study, which reported no significant differences in functional scores between treatment arms when all interventions yielded overall improvement [25]. Functional measures may be subject to a ceiling effect as patients in both groups begin to improve, further reducing the likelihood of detecting between-group differences. Adherence to home exercise is critical for rehabilitation. In our study, regular engagement in the exercise programs was maintained in both groups, with the app objectively recording all sessions, whereas three control participants lost their paper logs. At present, no widely established guideline exists regarding the optimal daily exercise duration for frozen shoulder. Consistent with this, our findings revealed no clear dose–response relationship between exercise frequency and clinical improvement. Although the Defrozen app group achieved greater gains in abduction and internal rotation, our analyses did not demonstrate a significant association between recorded exercise frequency and outcome improvements. These findings suggest that recorded exercise volume alone may not fully explain the observed differences between the groups. However, whether the app improved exercise quality could not be determined because exercise performance was not directly assessed. Similar mobile applications have incorporated animated exercise simulations and biofeedback to support frozen shoulder rehabilitation [35]. The integrated visual demonstrations may have facilitated participants’ understanding and execution of the prescribed exercises. However, exercise accuracy, kinesiophobia, and motor learning were not directly assessed; therefore, these potential mechanisms remain hypothetical.

This study has several limitations. First, the small sample size reflects its pilot nature and limits the generalizability of findings, although significant between-group differences favoring the app-guided group were still observed. Second, the four-week follow-up, constrained by budget considerations, could not capture long-term effects or potential dose–response relationships. Third, although ROM, pain, and patient-reported functional outcomes were assessed, instrumented biomechanical assessment was not included. Therefore, the mechanisms underlying the observed ROM improvements may be examined in future studies with longer follow-up. Fourth, the partial loss of adherence data in the control group highlights the limitations of traditional self-report monitoring. Future larger-scale trials with extended follow-up are warranted to validate these findings and to evaluate the sustainability of app-based benefits over time.

## 5. Conclusions

In this pilot randomized controlled trial, a four-week mobile app-based home exercise program (Defrozen app) was associated with greater short-term improvements in shoulder abduction and internal rotation compared with a conventional pamphlet-based program in patients with frozen shoulder following corticosteroid injection. Both groups achieved meaningful gains in pain relief and functional outcomes. Although these preliminary findings suggest that an mHealth-supported approach may add value to shoulder rehabilitation, larger and adequately powered randomized controlled trials with longer follow-up periods are needed to confirm these findings and clarify the potential role of app-assisted home exercise in the management of frozen shoulder.

## Figures and Tables

**Table 1:** Baseline characteristics of participants.

Characteristic	Experimental Group (*n* = 14)	Control Group (*n* = 7)	*p*-Value
Age (years), mean (SD)	54.64 (6.43)	56.14 (7.34)	0.636
Gender (Male:Female)	6:8	3:4	1.000
Dominant hand (Right:Left)	4:10	3:4	0.638
Range of motion of shoulder			
Flexion (°)	117.86 (17.35)	121.43 (27.85)	0.639
Extension (°)	43.36 (7.53)	39.71 (10.44)	0.370
Abduction (°)	82.14 (29.20)	96.29 (33.22)	0.165
Internal Rotation (°)	36.64 (23.70)	38.14 (26.03)	0.896
External Rotation (°)	54.64 (19.93)	38.14 (25.02)	0.184
Pain intensity and functional outcomes of the shoulder			
NRS (Pain)	6.07 (1.69)	7.14 (1.35)	0.161
OSS	30.57 (7.46)	20.29 (8.12)	0.009
QuickDASH score	36.00 (16.53)	51.29 (16.20)	0.059

Notes: Data represents the mean value (standard deviation). Abbreviations: NRS: Numerical Rating Scale; OSS: Oxford Shoulder Score; QuickDASH score: Quick Disabilities of the Arm, Shoulder, and Hand (QuickDASH) Score.

**Table 2:** Within-group pre- to post-intervention changes over 4 weeks.

Outcome Measure	Group	Pre Mean (SD)	Post Mean (SD)	Mean Difference (95% CI)	*p*-Value
Range of motion of shoulder					
Flexion (°)	Experimental	118.31 (17.97)	149.31 (18.79)	31.00 (20.35 to 41.65)	<0.001
	Control	121.43 (27.85)	143.00 (15.87)	21.57 (4.18 to 38.96)	0.023
Extension (°)	Experimental	43.23 (7.82)	55.69 (7.16)	12.46 (6.76 to 18.17)	<0.001
	Control	39.71 (10.44)	50.71 (6.68)	11.00 (0.95 to 21.05)	0.037
Abduction (°)	Experimental	81.54 (30.30)	139.23 (32.43)	57.69 (42.32 to 73.07)	<0.001
	Control	96.29 (33.22)	123.00 (22.92)	26.71 (9.16 to 44.27)	0.010
Internal Rotation (°)	Experimental	35.46 (24.23)	60.08 (19.08)	24.62 (12.95 to 36.28)	0.001
	Control	38.14 (26.03)	44.00 (22.80)	5.86 (−6.46 to 18.18)	0.289
External Rotation (°)	Experimental	55.38 (20.54)	73.77 (13.21)	18.38 (4.39 to 32.38)	0.014
	Control	38.14 (25.02)	80.00 (11.18)	41.86 (22.55 to 61.16)	0.002
Pain intensity and functional outcomes of the shoulder					
NRS	Experimental	6.00 (1.73)	2.23 (1.48)	−3.77 (−5.17 to −2.37)	<0.001
	Control	7.14 (1.35)	2.43 (2.23)	−4.71 (−6.62 to −2.81)	<0.001
OSS	Experimental	30.62 (7.76)	39.69 (6.87)	9.08 (3.16 to 14.99)	0.006
	Control	20.29 (8.12)	38.71 (3.09)	18.43 (9.97 to 26.89)	0.002
QuickDASH score	Experimental	36.00 (17.20)	14.77 (8.29)	−21.23 (−33.64 to −8.82)	0.003
	Control	51.29 (16.20)	18.14 (9.44)	−33.14 (−45.38 to −20.90)	<0.001

Notes: Data represents the mean value (standard deviation). Abbreviations: NRS: Numerical Rating Scale; OSS: Oxford Shoulder Score; QuickDASH score: Quick Disabilities of the Arm, Shoulder, and Hand (QuickDASH) Score.

**Table 3:** Between-group post-intervention comparisons adjusted for baseline values (ANCOVA).

Outcome	Model *p*-Value	R^2^	*p*-Value (Group) Partial η^2^ (95% CI)
Range of motion of the shoulder			
Flexion	0.017	0.382	0.271; 0.071 (0.000–0.364)
Extension	0.234	0.157	0.212; 0.090 (0.000–0.388)
Abduction	0.002	0.531	0.023; 0.269 (0.002–0.554)
Internal Rotation	<0.001	0.579	0.020; 0.279 (0.005–0.562)
External Rotation	0.329	0.123	0.180; 0.103 (0.000–0.404)
Pain intensity and functional outcomes of the shoulder			
NRS	0.845	0.020	0.967; 0.000 (0.000–0.032)
OSS	0.919	0.010	0.873; 0.002 (0.000–0.162)
QuickDASH score	0.730	0.036	0.581; 0.018 (0.000–0.269)

Abbreviations: ANCOVA: Analysis of Covariance; NRS: Numerical Rating Scale; OSS: Oxford Shoulder Score; QuickDASH score: Quick Disabilities of the Arm, Shoulder, and Hand (QuickDASH) Score.

**Table 4:** Regression models of difference scores (post−pre) with exercise time, baseline, and group as predictors.

Outcome	Model *p*-Value	R-Squared	Exercise Time Coef (*p*-Value)	Baseline Coef (*p*-Value)	Group (App vs. Pamphlet) Coef (*p*-Value)
Range of motion of the shoulder					
Flexion	0.092	0.359	+1.519 (0.627)	−0.519 (0.030)	−11.211 (0.241)
Extension	0.004	0.609	−1.803 (0.151)	−0.806 (0.001)	−4.344 (0.273)
Abduction	0.077	0.377	+1.862 (0.682)	−0.338 (0.117)	−29.185 (0.050)
Internal rotation	0.008	0.556	−2.881 (0.301)	−0.568 (0.004)	−19.534 (0.035)
External rotation	<0.001	0.752	+0.402 (0.893)	−0.873 (0.001)	+5.152 (0.555)
Pain intensity and functional outcomes of the shoulder					
NRS	0.001	0.674	−0.306 (0.260)	−1.075 (<0.001)	−0.158 (0.858)
OSS	0.001	0.671	−0.799 (0.519)	−0.967 (<0.001)	+0.612 (0.884)
QuickDASH score	<0.001	0.821	−0.958 (0.583)	−0.990 (<0.001)	+1.463 (0.801)

Notes: Each row represents a regression of the difference score (post–pre) on exercise time (continuous), baseline measure, and groups (experimental vs. control). Abbreviations: Coef: Coefficient; NRS: Numerical rating scale; OSS: Oxford Shoulder Score; QuickDASH score: Quick Disabilities of the Arm, Shoulder, and Hand Score.

## Data Availability

The data are available for research purposes upon request to the corresponding author.

## Abbreviations

The following abbreviations are used in this manuscript: Frozen shoulder, FS; range of motion, ROM; randomized controlled trial, RCT; numeric rating scale, NRS; Oxford Shoulder Score, OSS; Quick Disabilities of the Arm, Shoulder, and Hand score, QuickDASH score; activities of daily living, ADL; World Health Organization, WHO; electronic health, eHealth; mobile health, mHealth; analysis of covariance, ANCOVA.
